# Right atrial pressure and Guyton’s approach to fluid management

**DOI:** 10.1186/s13613-024-01402-2

**Published:** 2024-12-18

**Authors:** S. Madger

**Affiliations:** Critical Care, Professor of Medicine and Physiology, McGill University Heath Centre, 1001 Decarie Blvd, Montreal, Quebec H4A 3J1 Canada

I would like to thank Passos et al. [1] for their kind comments on my review on the use of Guyton physiology for fluid management [2]. However, they were concerned that I over-stated the value of right atrial pressure (RAP). This was a useful comment because it indicates a common confusion over terms and allows me to provide more precision for the term RAP.  I need you to insert this reference (it is the letter that I am responding to)

Passos et al. state: “*One key challenge lies in the assumption that RAP accurately reflects central venous pressure and thus venous return”.* RAP is a pressure and venous return is flow per minute and thus RAP does not *reflect* venous return. RAP indicates the following:

1. How well cardiac function is matching the venous return *function*. It does not indicate the magnitude of venous flow or cardiac output. RAP is normally below zero in the upright posture and cardiac output is obviously normal. However, RAP also could be zero because of a severe hemorrhage and inadequate filling of the heart. In that case, the heart is handling what it gets but it is not getting enough to put out!
2. RAP indicates the of upstream risk of tissue congestion [3]. When RAP relative to atmospheric pressure is high (“high” is likely greater than 10 mmHg), capillary filtration is increased, whether RAP is elevated because of high pleural pressure, high abdominal pressure, high blood volume or other factors, and whether cardiac output and venous return are high or low.
3. RAP relative to atmosphere is what matters for venous return, but RAP relative to pleural pressure, that is transmural RAP, is what matters for Starling’s law and cardiac output [3, 4]. Which one to use depends upon the question. RAP as commonly measured can be high because of a high pleural pressure or tamponade and be underfilled. Giving more volume will increase cardiac output but also increase tissue congestion.
4. It also is safe to say that if cardiac output increases and RAP falls, the primary hemodynamic change was an increase in pump function; alternatively, if cardiac output rises with a rise in RAP the primary hemodynamic change was a rise in circuit factors that increased venous return.

Passos et al. state that variations in in vascular compliance (presumably they meant capacitance), intra-abdominal pressures, and positive pressure ventilation, all can significantly alter the relationship between RAP and true venous return complicating the interpretation of hemodynamic status. True, detailed hemodynamics are complicated, but they are not in a vacuum. If there has been a change in RAP with a change in cardiac output, that is not a static RAP measure but rather a change in time, which is a “dynamic change”. If cardiac output (and thus venous return) fell with a fall in RAP, then look for a volume loss, increased capacitance by a drug, or increased venous resistance. If PEEP or tidal volumes were increased and RAP rises with a fall in cardiac output, then perhaps RV function was decreased by the increased pulmonary load and venous return decreased by the decreased pressure difference from the upstream veins. Cardiac output does not have to be measured; a clinical impression of a fall in output, i.e. cold extremities, decreased consciousness, or a fall in central venous saturation can help.

The normal range of RAP is small and measurements must be done accurately and consistently the same way [4]. RAP should be measured at the Z point, which is at the base of the ‘c’ wave at end-expiration (or ‘s’ wave on the QRS), whether the subject is breathing spontaneously or breathing with mechanical support, and forced expiratory efforts ruled out.

Finally, RAP does not and will not have a “predictive” value acutely. It indicates what is happening at that time between the cardiac and return functions. If one learns how to use RAP properly, the pattern of change can be a simple and easy adjunct to clinical decision making. As a final word, a sustained high RAP is associated with worse outcomes and does have a predictive value [2].

## Data Availability

Not applicable.

## References

[1] Passos et al. https://doi.org/10.1186/s13613-024-01416-w.

[2] Magder S. The use of Guyton’s approach to the control of cardiac output for clinical fluid management. Ann Intensive Care. 2024;14(1):105. 38963533 10.1186/s13613-024-01316-z PMC11224168

[3] Pesenti A, Slobod D, Magder S. The forgotten relevance of central venous pressure monitoring. Intensive Care Med. 2023;49(7):868–70. 37294343 10.1007/s00134-023-07101-z

[4] Magder S. Right Atrial Pressure in the Critically Ill: How to Measure, What Is the Value, What Are the Limitations? Chest. 2017;151(4):908–16. 27815151 10.1016/j.chest.2016.10.026

